# Survie au cancer du sein à Rabat (Maroc) 2005-2008

**DOI:** 10.11604/pamj.2016.25.144.10402

**Published:** 2016-11-11

**Authors:** Nada Bennani Mechita, Mohammed Adnane Tazi, Abdelouahed Er-Raki, Mustapha Mrabet, Asma Saadi, Noureddine Benjaafar, Rachid Razine

**Affiliations:** 1 Laboratoire de Santé Publique, Faculté de Médecine et de Pharmacie, Université Mohammed V, Rabat, Maroc; 2 Registre des Cancers de Rabat/Institut National d’Oncologie, Rabat, Maroc; 3 Laboratoire d’Hygiène du Milieu, Hôpital Militaire d’Instruction Mohammed V, Rabat, Maroc

**Keywords:** Cancer, sein, survie, Maroc, Cancer, breast, survival, Morocco

## Abstract

**Introduction:**

Le cancer du sein représente un problème de santé publique au Maroc. L’objectif de ce travail était d’estimer le taux de survie au cancer du sein chez les patientes habitant la ville de Rabat.

**Méthodes:**

Etude pronostique réalisée chez les patientes diagnostiquées pour cancer du sein de 2005 à 2008, habitant la ville de Rabat et enregistrées au registre des cancers de Rabat. La date d’inclusion dans l’étude correspondait à la date de confirmation histologique du cancer. L’estimation de la survie a été réalisée par la méthode de Kaplan Meier, et la comparaison entre les différentes classes d’une variable a été réalisée par le test de log rank. L’étude des facteurs associés à la survie a été effectuée par le modèle de Cox.

**Résultats:**

Durant la période d’étude 628 cas de cancer du sein ont été collectés. Le pourcentage de décès était de 19,9%. La survie globale à un an était de 97,1%, elle était de 89,2% à 3 ans et de 80,6 % à 5 ans. En analyse multivariée la survie au cancer du sein était statistiquement moins bonne chez les patientes âgées de plus de 70 ans (p<0,001), ayantune grande taille de tumeur (p<0,001), un stade avancé d’adénopathies (p=0,007), présentant des métastases (p<0,001) et non traitées par hormonothérapie (p=0,002).

**Conclusion:**

Une grande taille de la tumeur et la présence de métastases sont des facteurs de mauvais pronostic du cancer du sein d’où la nécessité de renforcer les programmes de dépistage.

## Abstract (en)

**Introduction:**

Breast cancer is a public health problem in Morocco. This study aims to estimate the survival rate for patients with breast cancer living in Rabat.

**Methods:**

We conducted a prognostic study of female patients with breast cancer diagnosed during 2005-2008, living in Rabat and whose data were recorded in the Rabat Cancer Registry. The date of inclusion in this study corresponded with the date on which cancer was histologically confirmed. Survival rate was estimated using the Kaplan-Meier method and the comparison between the different classes of a variable was made using the log rank test. The study of factors associated with survival was performed using the Cox model.

**Results:**

During the study period 628 cases of breast cancer were collected. Mortality rate was 19.9%. Overall 1-year survival rate was 97.1%, 89.2% at 3 years and 80.6% at 5 years. In multivariate analysis, breast cancer survival was statistically lower in patients over 70 years of age (p <0.001) with large tumor size (p < 0.001), advanced-stage adenopathies (p = 0.007), metastases (p < 0.001) and not using hormone therapy (p = 0.002).

**Conclusion:**

Large tumor size and metastases are poor prognostic factors in breast cancer, hence the need to strengthen screening programs.

## Introduction

Le cancer du sein représente un réel problème de santé publique au niveau mondial, c’est le deuxième cancer en terme de fréquence dans le monde (après le cancer du poumon), touchant 1.7 millions de cas en 2012 ce qui représente 11.9% du total des cancers et le cancer le plus fréquemment diagnostiqué chez la femme [1]. Le cancer du sein est également la première cause de décès chez la femme [1].

Les taux de survieau cancer du sein à 5 ans sont extrêmement variables d’un pays à l’autre, allant de 80% ou plus en Amérique du Nord, en Suède et au Japon à près de 60% dans les pays à revenu intermédiaire, et à moins de 40% dans les pays à faible revenu [2]. Au Maroc, le cancer du sein constitue un véritable problème de santé publique, il représente le premier cancer chez la femme (36.1% des cancers chezles femmes) [3]. Selon les données marocaines émanant des deux registres de cancer de population [4, 5], l’incidence annuelle brute du cancer du sein chez la femme en 2007 était de 54,7pour 100 000 à Rabat et 39.9 pour 100 000 femmesau grand Casablanca [4]. L’incidence standardisée sur la population mondiale était de 48,1 et 38,6 pour 100000 femmes respectivement à Rabat et au grand Casablanca [4, 5].

Une étude de survie au cancer du sein a été réalisée chez 74 femmes jeunes dans le nord-est du Maroc et a objectivé une survie globale à 3 ans de 87.8% [6]. Mais jusqu’à présent aucune étude de survie au cancer du sein n’a été réalisée sur une base de population au Maroc. L’objectif de ce travail, était d’estimer le taux de survieet les facteurs pronostiques du cancer du sein chez les patientes résidant à Rabat.

## Méthodes

### Lieu et schéma d’étude

Cette étude a été réalisée au niveau du registre des cancers de Rabat à l‘institut National d’Oncologie (INO). C’est une structure qui enregistre de manière exhaustive et permanente tous les nouveaux cas de cancer diagnostiqués à partir de l’année 2005 chez les personnes résidant administrativement à la ville de Rabat. Il s’agit d’une étude pronostique incluant toutes les patientes habitant Rabat et diagnostiquées pour cancer du sein entre 2005 et 2008.

### Recueil des données

Les variables étudiées étaient: l’Age, la date de diagnostic, le type histologique,la taille de la tumeur (T) (T1≤2cm, 2cm 5cm et T4: extension à la paroi thoracique ou à la peau),le stade d’adénopathies (N) (N0: pas d’envahissement ganglionnaire régional, N1 :envahissement de 1 à 3 ganglions axillaires et/ou envahissement de la chaine mammaire interne détecté sur ganglion sentinelle sans signe clinique, N2: envahissement de 4 à 9 ganglions axillaires ou envahissement des ganglion mammaires internes homolatéraux suspects, en l’absence d’envahissement ganglionnaire axillaire, N3: envahissement d’au moins 10 ganglions axillaires ou des ganglions sous-claviculaires ou des ganglions mammaires internes homolatéraux avec envahissement ganglionnaire axillaire), la présence (M1) ou non (M0) de métastases, le stade de la tumeur selon la classification TNM [7] et le type de traitement: chirurgie, radiothérapie, chimiothérapie, ou hormonothérapie qui est indiquée chez les patientes ayant des récepteurs hormonaux positifs. Le statut vitaldes patientes a été recherché au niveau des structures de prise en charge des malades via leur médecin traitant (à partir des dossiers médicaux ou par contact téléphonique) et à partir des données du registre de décès de la ville de Rabat qui enregistre tous les cas de décès qui surviennent dans cette ville. La date d’inclusion dans l’étude a été considérée comme la date de confirmation histologique du cancer et la date de point a été fixée au 01/07/2014.

### Analyse statistique

Nous avons réalisé une analyse descriptive de la population d’étude, où les variables quantitatives ont été représentées en moyenne et écart-type et les variables qualitatives en effectif et pourcentage. Nous avons ensuite comparé les différentes variables entre les sujets perdus de vue et les sujets ayant complété le suivi. Pour la comparaison des variables qualitatives nous avons utilisé le test de chi 2 et pour la comparaison des variables quantitatives nous avons utilisé le test de student.

L’estimation de la survie a été réalisée par la méthode de Kaplan Meier, et la comparaison entre les différentes classes d’une variable a été réalisée par le test de Log Rank. L’étude des facteurs associés à la survie a été effectuée par le modèle de Cox, les variables associéessignificativement à la survie en analyse univariée ont été introduite dans le modèle multivarié. Le seuil de significativité a été fixé à 0,05. L’analyse des données a été effectuée par le logiciel SPSS version 13.0.

## Résultats

Durant la période d’étude 628 nouveaux cas de cancer du sein ont été enregistrés, ce qui représente une fréquence de 37,7% de tous les cancers diagnostiqués chez les femmes durant cette période. L’incidence brute moyenneannuelle de 2005 à 2008 était de 46,6 pour 100000 habitants et l’incidence standardisée sur la population mondiale était de 41,5 pour 100000 habitants.

La moyenne d’âge était de 51,1 ±11,2 ans. La tumeur a été confirmée microscopiquement dans 99,8% des cas (n=626) et le type histologique le plus fréquemment observé était le carcinome canalaire infiltrant (83,9% ; n=527). Le cancer était au stade I chez 89 patientes (14,2 %) et au stade II chez 269 patientes (42,8%). Seules 40 patientes (6,4%) étaient au stade IV et le stade n’a pas été déterminé chez 68 patientes (10,8%). La chirurgie a été réalisée chez 567 patientes (90,3%) ,la radiothérapie chez 464 patientes (73,9%), la chimiothérapie chez 493 patientes (78,5%) et l’hormonothérapie chez 316 patientes (50,3%). La comparaison de ces différentes variables entre le groupe de sujets ayant complété le suivi et les patients perdus de vue n’a montré aucune différence statistiquement significative.

Le pourcentage de décès était de 19,9% (n=125) et le pourcentage de perdus de vus à la date de point était de 32,2% (n=202).La durée moyenne de suivi était de 98,23 mois.

**Figure 1:**
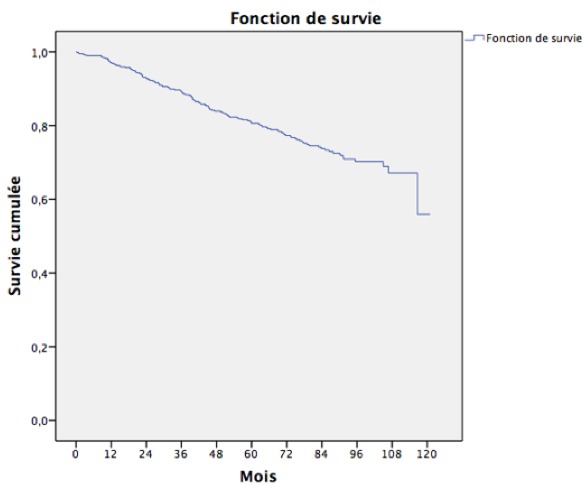
Survie globale au cancer du sein chez les patientes habitant Rabat, 2005-2008

**Figure 2:**
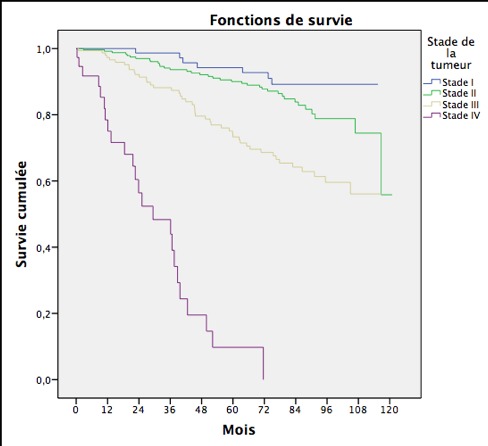
Survie au cancer du sein selon le stade de la tumeur, Rabat 2005-2008

La survie globale était de 97,1% à un an, 89,2% à 3 ans et 80,6% à 5 ans (Tableau 1) et (Figure 1). La survie à 5ans était plus basse chez les patientes âgées de plus de 70ans (55,6%) par rapport aux femmes âgées de moins de 70 ans (71,6%) (p<0,001). Les femmes ayant une tumeur au stade IV avaient une survie à 5 ans moins bonne (9,8%) que les femmes ayant une tumeur au stade I (94,2%;(p<0,001)) (Figure 2).Les patientes ayant bénéficié d’une hormonothérapie avaient une meilleure survie par rapport aux patients n’en ayant pas bénéficié (86,6% vs 73,1%; (p<0,001)) (Tableau1).

**Tableau 1:** Cancer du sein: survie globale et selon les caractéristiques des patientes habitant Rabat, 2005-2008

	Effectif	Survie à 1an %	Survie à 3ans %	Survie à 5ans %
Survie globale	628	97,1	89,2	80,6
Age				
<40 ans	79	97,2	81,8	78,0
40-54 ans	337	98,9	91,6	83,3
55-69 ans	166	95,5	92,4	83,1
> 70 ans	44	89,2	71,6	55,6
Taille de la tumeur α				
T1	138	100,0	97,4	93,5
T2	303	98,2	92,9	86,9
T3	77	96,7	87,2	64,5
T4	59	84,1	54,0	40,5
Adénopathies β				
N0	270	98,7	95,4	91,3
N1	172	98,0	86,3	76,6
N2	89	93,9	86,9	73,8
N3	42	100,0	82,6	62,1
Métastases ¥				
M0	530	98,8	92,7	85, 7
M1	40	78,5	48,4	9,8
StadeΩ				
I	89	100 ,0	98,6	94,2
II	269	99,2	93,6	90,5
III	162	97,3	88,2	73,3
IV	40	78,5	48,4	9,8
Hormonothérapie				
Oui	316	99,7	94,0	86,6
Non	312	94,7	83,2	73,1

α Inconnu pour 51 patientes

β Inconnu pour 55 patientes

¥ Inconnu pour 58 patientes

Ω Inconnu pour 68 patientes

En analyse univariée, la survie au cancer du sein était statistiquement associée à l’âge, à la taille de la tumeur, au stade d’adénopathies, à la présence de métastases et à l’hormonothérapie (Tableau 2). En analyse multivariée, le risque de décès augmentait avec l’âge (RR=2,77 pour âge? 70ans; âge<40ans étant la classe de référence), avec la taille de la tumeur (RR=6,38 pour la taille T4; la taille T1 étant la classe de référence) et avec le stade d’adénopathies (RR=3,45 pour les N3 ;N0 étant la classe de référence). Le risque de décès était également plus élevé chez les patientes ayants des métastases (RR= 5,24) et chez les patientes non traitées par hormonothérapie (RR=1,88).

**Tableau 2:** Etude des facteurs associés à la survie au cancer du sein en analyse uni et multivariée par le modèle de Cox, Rabat 2005-2008

	Analyse univariée			Analyse multivariée		
	RR	IC 95%	P	RR	IC 95%	P
Age			**<0,001**			**<0,001**
<4O ans	1,00			1,00		
40-54 ans	0,56	[0,33- 0,93]	0,047	0,56	[0,33- 0,95]	0,032
55-69 ans	0,64	[0,36- 1,12]	<0,001	0,70	[0,40- 1,25]	0,230
> 70 ans	1,89	[1,01- 3,54]	<0,001	2,77	[1,44- 5,31]	0,002
Taille de tumeur			**<0,001**			**<0,001**
T1	1,00			1,00		
T2	2,37	[1,24- 4,54]	**0,009**	1,92	[0,99- 3,74]	0,054
T3	5,23	[2,51- 10,86]	**<0,001**	3,38	[1,56- 7,31]	0,002
T4	13,72	[6,85- 27,47]	**<0,001**	6,38	[2,97-13,69]	<0,001
Adénopathies			**<0,001**			**0,007**
N0	1,00			1,00		
N1	2,33	[1,47- 3,70]	<0,001	1,79	[1,11- 2,88]	0,016
N2	2,32	[1,30- 3,95]	0,004	1,60	[0,90-2,84]	0,111
N3	4,40	[2,46- 7,88]	<0,001	3,45	[1,87- 6,38]	<0,001
Métastases			**<0,001**			**<0,001**
M0	1,00			1,00		
M1	14,73	[9,01- 24,11]		5,24	[2,99-9,17]	
Hormonothérapie			**<0,001**			**0,002**
Oui	1 ,00			1,00		
Non	2,41	[1,68- 3,46]		1,88	[1,24-2,67]	

## Discussion

Dans notre population d’étude, la survie globale à 5 ans au cancer du sein était de 80,6 % ce qui estplus bas que les taux retrouvés dans certaines études de pays développés (86,6 à 89,7%) [8–10] et plus élevés que le taux observé au Vietnam [11]. Le taux de survie plus élevé dans les pays plus développés pourrait être expliqué par la présence de programme de dépistage et d’infrastructure de diagnostic et de traitement plus développées.

Les femmes âgées entre 40 et 69 ans au moment du diagnostic avaient la meilleure survie par rapport aux autres tranches d’âge, avec un taux de survie à 5 ans à 83%. Ces résultats concordent avec les résultats d’une étude qui a objectivé que la survieau cancer du sein à 5 et 10 ans était était meilleure chez les femmes âgées de 46-50 au moment du diagnostic [12].

En analyse multivariée, un âge de plus de 70 ans était un facteur de mauvais pronostic.Cet excès de mortalité après 70 ans peut être dû à la réduction normale de l´espérance de vie avec l’âge.La comparaison de la survie relative par classe d’âge aurait pu confirmer ou infirmer cette hypothèse. En effet une étude réalisée en France a objectivé que la différence entre les taux de survie relative chez les différentes classes d’âge n’étaient pas statistiquement significatives [13].

L’augmentation de la taille de la tumeur, le stade avancé d’adénopathie et la présence de métastases étaient égalementassociés à un mauvais pronostic, ce qui concorde avec plusieurs études [14–16]. Alors que d’autres études ont trouvé que le nombre de ganglions atteints n’avait aucune influence sur la survie [13].

Le traitement par hormonothérapie était égalementassocié à une meilleure survie à 5ans. Les patientes n’ayant pas bénéficié de l’hormonothérapie avaient 2 fois plus de risque de décéder que les patientes en ayant bénéficié. Ces résultats concordent avec les résultats rapportés dans de précédentes études [17, 18]. L´effet du traitement hormonal est le plus souvent associé à la présence de récepteurs hormonaux. Une métaanalyse des essais randomisés a démontré que le tamoxifène était efficace chez les patientes ayant des récepteurshormonaux positifs par rapports aux patientes ayant des récepteurs hormonaux négatifs [18]. De plus, une étude a objectivéque la présence de ces récepteurs hormonaux est associée à une meilleure survie au cancer du sein [19].

Une limite doit être prise en compte lors de l´interprétation des résultats de cette étude. La proportion élevée de patients perdus de vue (32,2%) peut avoirentrainé un biais de sélection, bien que leurs caractéristiques ne diffèrent pas de celles des personnes ayant eu un suivi complet. Les données relatives à l’état de survie des patientes ont été recherchées auprèsdes médecins traitants à partir des dossiers médicaux ou par contact téléphoniquedes patients et à partir des données du registre de mortalité de Rabat qui enregistre de manière continue et exhaustive tous les décès survenant à la ville de Rabat. Seuls les sujets décédés dans une autre ville ou àun autre pays pourraient ne pas figurer dans ce registre, ce qui représente une proportion minime. Il y a donc peu de chance que ces patients perdus de vu soient décédés.

Malgré ces limites, c´est la première analyse de survie ducancer du sein réalisée au Maroc à partir des données d’un registre de cancer de population.

## Conclusion

La taille élevée de la tumeur et la présence de métastases sont des facteurs de mauvais pronostic du cancer du sein d’où la nécessité de renforcer le programme dépistage du cancer du sein.

### Etat des connaissances actuelle sur le sujet

- Le cancer du sein est le premier cancer chez la femme dans le monde;
- Il représente un problème de santé publique au Maroc.

### Contribution de notre étude à la connaissance

- C’est la première étude d’analyse de survie au cancer du sein surune base de population au Maroc.

## Remerciements

Nous remercions l’ensemble des Directeurs des Hôpitaux publiques et des cliniques privées ainsi que les chefs de service de l’ensemble des unités où nous avons collecté les données et le personnel du Bureau municipal d’hygiène de Rabat. Nous remercions également les résidents du service de radiothérapie à l’INOpour leur contribution à la collecte des données et au personnel des archives de l’INO pour avoir mis à notre disposition les dossiers médicaux. Enfin nous remercions l’équipe du registre de cancer de Rabat.
